# Can extended reality in the metaverse revolutionise health communication?

**DOI:** 10.1038/s41746-022-00682-x

**Published:** 2022-09-02

**Authors:** Adéla Plechatá, Guido Makransky, Robert Böhm

**Affiliations:** 1 Department of Psychology, University of Copenhagen, Copenhagen, Denmark; 2 Faculty of Psychology, University of Vienna, Vienna, Austria; 3 Copenhagen Center for Social Data Science (SODAS), University of Copenhagen, Copenhagen, Denmark

**Keywords:** Human behaviour, Communication

## Abstract

In the metaverse, users will actively engage with 3D content using extended reality (XR). Such XR platforms can stimulate a revolution in health communication, moving from information-based to experience-based content. We outline three major application domains and describe how the XR affordances (presence, agency and embodiment) can improve healthy behaviour by targeting the users’ threat and coping appraisal. We discuss how health communication via XR can help to address long-standing health challenges.

The predicted arrival of the metaverse has given rise to heated public debate. Critics of the metaverse argue that companies’ economic self-interest is primarily responsible for the hype surrounding the metaverse and that such a platform would be difficult to regulate. Some critics argue that the metaverse poses a ‘terrifying danger to humanity’^1^, potentially leading to a surge in harassment and manipulation^2^. However, others contend that the metaverse could ‘unleash amazing creativity and open up new frontiers and horizons’^3^, providing a platform for new tools to learn, socialise and collaborate in cyberspace.

Adding to the debate, we focus on the tremendous potential of the metaverse to revolutionise health communication. Public access to the Internet marked the first revolution in health communication by diminishing the role of the general practitioner as the sole authority and source of health-related information. The metaverse can stimulate a second revolution, allowing users to engage actively with tailored health-related information from an interactive first-person perspective. In other words, we will witness a transformation from information- to experience-based health communication. We argue that by creating theory-driven and evidence-based health communication content using immersive technologies, the metaverse can significantly promote individual and public health. In addition, we outline how future efforts in this domain can address long-standing challenges to public health and point to new challenges arising from this transformation.

## What is the metaverse?

The term metaverse was used for the first time by Neal Stephenson in his novel *Snow Crash*^4^, where he describes people using avatars to interact with each other in virtual worlds. This vision resembles virtual worlds that we already know, such as Second Life, Fortnite or VRChat. The metaverse adds extensive use of 3D graphics to this, allowing users to permanently access online content using extended reality (XR)^5^. XR technologies encompass a wide spectrum of immersive technologies, from augmented reality (AR) to mixed reality (MR) to virtual reality (VR). These technologies differ in how much of the outside world is included in the experience, with VR at the end of the immersive spectrum, providing users with a virtual experience that almost completely shuts out the external environment. Although XR technologies have been available for many decades, the metaverse would allow us to bring them together and make them accessible to everybody anywhere, all the time.

## How does XR in the metaverse facilitate effective health communication?

**Fig. 1:**
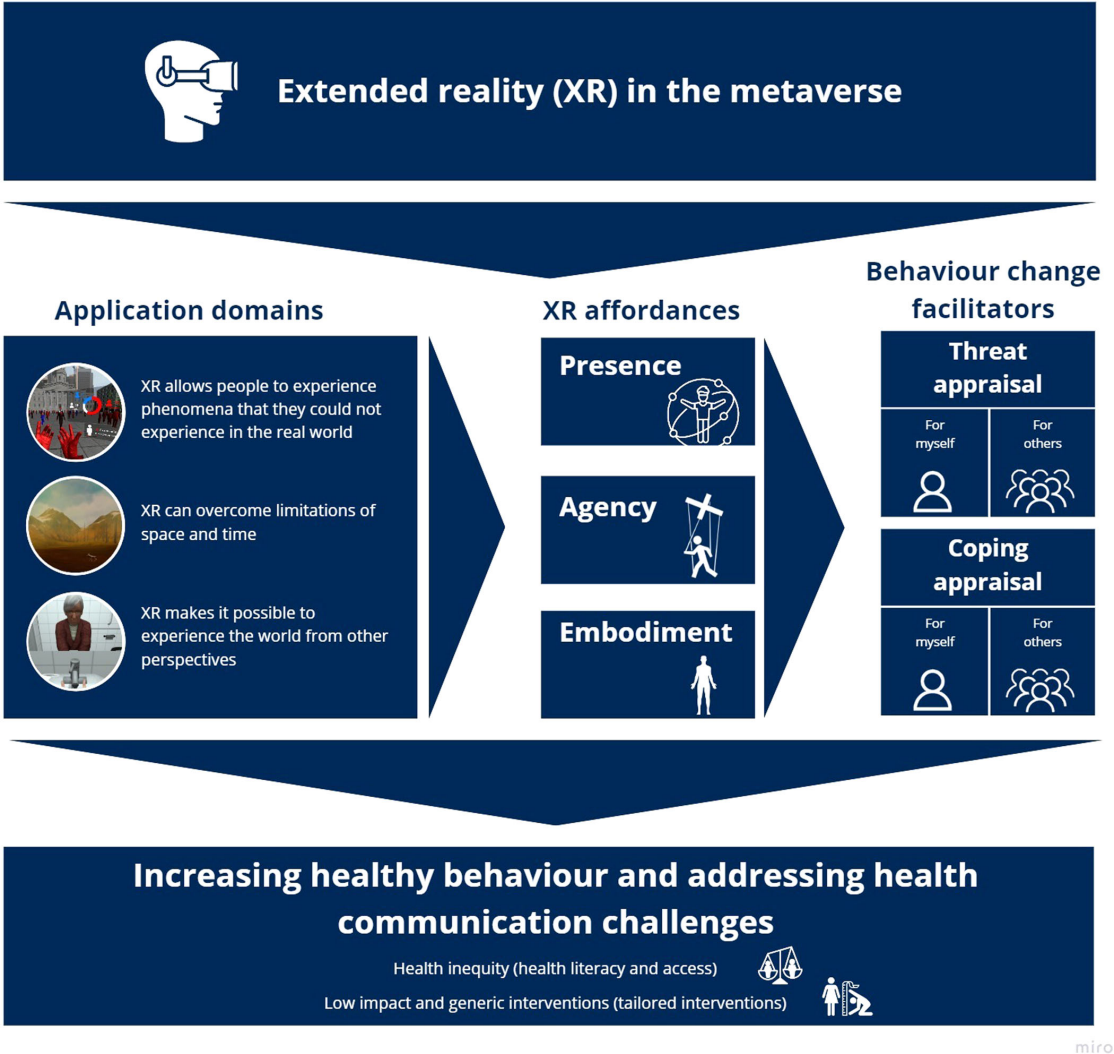
Extended reality (XR) in health communication. *Note*: The figure depicts three major application domains and XR affordances and shows how they can improve healthy behaviour by targeting self- and other-directed threat and coping appraisal.

There is abundant evidence indicating that XR, in particular VR, can create realistic experiences that produce genuine emotional, cognitive, social and behavioural reactions^6,7^. The content presented and experienced in XR can be modified in such a way that it is particularly useful for effective health communication, with three main application domains (Fig. 1). First, XR allows people to experience phenomena that they could not experience in the real world (e.g., visualising how otherwise unobservable viruses or bacteria spread)^8^. Second, XR can overcome limitations of space and time by communicating the negative consequences of unhealthy behaviours that are temporarily distant from the present behaviour, such as obesity or smoker’s lungs^9,10^. Third, XR makes it possible to experience the world from other perspectives, including those of vulnerable individuals, thereby eliciting empathy and compassion^11^.

According to Protection Motivation Theory (PMT)^12^, a prominent and widely adopted theoretical framework for disease prevention and health promotion, successful health communication requires improving *threat appraisal* and *coping appraisal. Threat appraisal* encompasses two aspects: *threat severity* (i.e., perceived level of severity of the threat to health) and *vulnerability* (i.e., perceived level of susceptibility to the threat). *Coping appraisal* encompasses *response efficacy* and *self-efficacy*. *Response efficacy* refers to an individual’s belief as to whether a recommended coping behaviour will effectively reduce a health-related threat; *self-efficacy* describes the confidence that the individual has in his/her ability to perform the recommended behaviour. Compared to cyberspace and social media accessed using standard media (i.e., computers or mobile devices), which already play a crucial role in health communication^13^, immersive XR has specific affordances of *presence*, *agency* and *embodiment*^14^. These affordances can be addressed in health communication via the metaverse, for instance, by providing immersive environments for patient support groups, expert-moderated health communities^13^ or health-aware pre-programmed agents and immersive health messages to facilitate health-related behaviour change more effectively than standard media (Fig. 1)^15^. Although these XR affordances are empirically related^16^, each affordance can conceptually be linked to specific health-related perceptions, that is, *threat* and *coping appraisal*, which we discuss below.

### Presence

According to PMT, low *threat appraisal* of temporally distant consequences of current unhealthy behaviours frequently explains people’s reluctance to adopt health-protective behaviours. This idea is supported by construal level theory^17^, which states that events, including threats, that are temporally, spatially or socially distant, are perceived as abstract and therefore as less relevant. As compared to the standard text, image or video interventions, XR technology can elicit higher presence, a feeling of being ‘there’ in the simulated environment^18^. Presence is comprised of three sub-dimensions: physical presence (i.e., place illusion), self-presence (i.e., perceived authenticity of our self-representation in the virtual environment) and social presence (i.e., perceived realness of virtual others)^19^. Being highly present in the virtual environment makes scenarios, such as experiencing the consequences of unhealthy behaviours^9^, feel more imminent and therefore increases *threat appraisal*^20^. Experiencing the consequences of behaviours is also crucial for *coping appraisal*, particularly *response efficacy*, as it allows the individual to vividly experience the impacts of such behaviours from a first-person perspective instead of reading about them.

Furthermore, as we are social creatures, and others largely influence our behaviour, high social presence, which results in increased social influence^21^, plays an important role in behavioural change. In health communication, avatars are often used to exemplify health consequences and influence our decisions. We can be affected by the presence of others via social norms and conformity^22^, such as the presence of ‘health-aware’ programmed agents (e.g., interacting with an agent who engages in exercising and healthy eating could influence the user to do the same). In a similar vein, *self-efficacy*, which plays a major role in adopting new behaviour and closing knowledge-behaviour gaps^23^, can be enhanced not only by our past experience but also by observing others similar to ourselves succeeding in the desired action^24^.

### Agency

According to Bandura, personal experience of success is the most influential source of *self-efficacy*^24^. As compared to immersive videos or more fixed scenarios that do not allow users to take their own courses of action, a highly interactive XR environment elicits the feeling of being in control of one’s actions (i.e., a sense of agency). Agency provides the user with a first-person interactive experience of success, i.e., mastery experience^14,24^, and is crucial for promoting *self-efficacy* and, in turn, the intention to adopt healthy behaviour. For example, Fox et al. showed that individuals who experienced positive effects of their own physical exercise on their virtual selves in an XR environment undertook more exercise in real life^25^.

### Embodiment

Although PMT and similar theories focus on the protection of the actors themselves, the health behaviours of individuals can provide social externalities to others, for example, by indirectly protecting others by increasing herd immunity via vaccination^26^. The XR affordance embodiment refers to the illusion of ownership of the virtual body^14^. The embodiment allows users to experience the world from someone else’s perspective, including someone of a different gender, race or species, and, in turn, influences our attitudes towards them^27^. The successful embodiment can promote positive health-related decisions by making the experience more personal, increasing the user’s perceived own *vulnerability* but also the vulnerability of others. For example, being embodied as a vulnerable person and therefore experiencing health threats from that person’s perspective, as well as the impact of others’ behaviour on the person’s health, can affect other-directed *threat* and *coping appraisal*, motivating the user to engage in behaviours that benefit not only their own health but that of others^8^. Due to such an embodied vivid perspective-taking experience, XR, particularly VR, has been labelled the ultimate ‘empathy machine’^11^. In line with this perspective, studies have shown that embodying users in the body of a person who is vulnerable to COVID-19 increases their collective responsibility to get vaccinated and, in turn, vaccination intention^8,28^.

## How can the metaverse address long-standing challenges in health communication?

Public health is plagued by several challenges, intensively discussed in the literature, that are difficult to overcome with standard health communication methods. Here, we outline how these challenges could be tackled by combining XR affordances with social aspects and the future accessibility of the metaverse.

Health inequity, which leads to unequal health outcomes and arises from economic, societal and environmental barriers, is one such challenge^29^. Two crucial factors contribute to health inequity: systematic differences in accessibility to evidence-based health information and the ability to process such information (health literacy)^29,30^. The metaverse could help address both of these factors. First, with the increasing accessibility and affordability of XR devices, individuals worldwide, including those in developing countries, could participate in the metaverse in the next decade. Second, as noted above, the metaverse represents a shift from information- to experience-based health communication. XR affordances provide visceral experiences that are easy to interpret and intuitively understandable. Therefore, XR can assist in the information processing of abstract health concepts. For instance, instead of processing complex scientific evidence about the relationship between (un)healthy eating, obesity and cardiovascular disease, users can be embodied in an avatar that allows the first-hand experience of this nexus. Such experience-based learning can overcome health inequity related to poor health literacy, something that has been shown to undermine the effectiveness of standard health communication methods^30^.

Another challenge in health communication is that current health campaigns targeting the general public typically have weak effects on actual health behaviours because people perceive them as unrelated and unengaging^31^. In contrast, there is increasing evidence that health interventions that are tailored specifically to a recipient’s characteristics and needs are more effective than generic messages^32^. Standard interventions usually allow customisation based only on broad characteristics, such as gender, race or age. In the metaverse, it would be possible to tailor health interventions based on multisensory health-related data, including body movements or health measures within or outside the metaverse, for instance, by linking virtual experiences to data from trackers or wearables. Such ‘dynamic tailoring’ of interventions rather than ‘static tailoring’ would allow interventions to be created and adapted specifically for individual users. Furthermore, it could improve *threat* and *coping appraisal* among population groups (e.g., young males) that are usually less responsive to standard health communication^32^.

## Conclusions and outlook

It is predicted that the metaverse will have a major influence on how we behave and interact, with 25% of us spending at least 1 h a day in the metaverse by 2026^33^. Despite the challenges arising from the potentially massive impact of the metaverse on our lives, it offers great potential for a paradigm shift in health communication. Indeed, current evidence from ‘lab-in-the-field’ experiments shows the promise of XR health communication in effectively improving healthy behaviour, even when self-administered^28^ or when administered in a non-laboratory environment, such as in public parks^8^ or afterschool^34^ or community settings^35^. Ultimately, the metaverse could revolutionise health communication by providing novel solutions to long-standing and pressing challenges in the field of public health.

By utilising the metaverse in health communication, new challenges arise. For instance, collecting and linking data within and outside the metaverse creates issues with data privacy. There is a need to find solutions to address these issues and develop guidelines and legislation on what and how information is used, including the problem of misinformation^36^. Lessons learned from classic social media are important and useful in this regard^13^. We also need to reach an agreement on ethical issues relating to the exploitation of the technological potential of the metaverse and our interactions within the metaverse. A useful step in this direction is the GuestXR project^37^. As part of this project, VR experts from academia and the private sector apply machine learning in XR to create virtual agents that can mediate information and social exchange in the metaverse and therefore limit harassment and conflicts in the metaverse.

Only when we find solutions to how health communication can be adapted to XR environments in ways that are both evidence-based and ethically responsible can XR platforms, such as the metaverse, truly have a positive impact on individual and public health.

### Reporting summary

Further information on research design is available in the Nature Research Reporting Summary linked to this article.

## Supplementary information

Reporting Summary

## Data Availability

This research does not report on novel original data.

## Code availability

This research does not rely on any code.
